# Blood microbiome signatures in the REM sleep behavior disorder–Lewy body disease continuum

**DOI:** 10.1007/s00702-025-02953-9

**Published:** 2025-06-04

**Authors:** Ryul Kim, Sujin Oh, Kyung Ah Woo, Cheol Min Shin, Kyoung Un Park, Jee-Young Lee

**Affiliations:** 1 Department of Neurology, Seoul Metropolitan Government - Seoul National University Boramae Medical Center, Seoul National University College of Medicine, Seoul, Korea; 2 Department of Laboratory Medicine, Seoul National University Bundang Hospital, Seoul National University College of Medicine, Seongnam, Korea; 3 Department of Internal Medicine, Seoul National University Bundang Hospital, Seongnam, Korea

**Keywords:** Lewy body, REM sleep, Parkinson, Blood, Microbiome

## Abstract

Although systemic inflammation triggered by alterations in microbiota from various body sites has been proposed as a potential mechanism underlying Lewy body diseases (LBDs), the association between the blood microbiome and LBDs remains uncertain. This study aimed to investigate the blood microbiome profiles across the REM sleep behavior disorder (RBD)–LBD continuum and to explore their potential as biomarkers reflecting disease phenotypes and clinical severity. Blood samples were collected from 106 patients across the RBD–LBD continuum, including 41 with isolated RBD (iRBD), 45 Parkinson’s disease with probable RBD, and 20 dementia with Lewy bodies with probable RBD, as well as from 94 healthy controls. All patients were evaluated with the Movement Disorder Society–Unified Parkinson’s Disease Rating Scale (MDS-UPDRS) and comprehensive neuropsychological tests. Microbiome taxonomic compositions were analyzed using 16 S rRNA metagenomic sequencing. Significant microbial shifts were observed in the RBD–LBD continuum group compared to controls, with reduced microbial alpha diversity and distinct beta diversity patterns. Specifically, the genus *Stenotrophomona*s was enriched, while the genera *Acetobacter*, *Enhydrobacter*, and *Lactobacillus* were depleted in the RBD–LBD continuum group. The combined model using these genera demonstrated high predictive accuracy for the RBD–LBD continuum, with the area under the receiver-operating-characteristic curve (AUC) of 0.970 (95% confidence interval [CI]: 0.950–0.980). This model also successfully distinguished the iRBD subgroup from controls, achieving an AUC of 0.956 (95% CI, 0.914–0.987). Alpha and beta diversity were significantly associated with MDS-UPDRS Parts I and II scores in the RBD–LBD continuum group. Our findings suggest that patients within the RBD-LBD continuum may share specific blood microbiome signatures.

## Background

Lewy body disorders (LBDs), including Parkinson’s disease (PD) and dementia with Lewy bodies (DLB), are characterized by the pathological aggregation of α-synuclein into Lewy bodies and Lewy neurites (Wakabayashi et al. 2013). Isolated REM sleep behavior disorder (iRBD) is currently recognized as the most robust prodromal marker of LBDs, with more than 80% of affected individuals eventually developing either a parkinsonism-first phenotype (PD) or a dementia-first phenotype (DLB) over a period of 10 years or more (Postuma et al. 2019). The shared presence of RBD prior to disease onset in PD and DLB suggests that the two disorders exist along a clinicopathological continuum. Supporting this notion, previous studies have shown that PD with RBD (PDRBD) is linked to greater cognitive impairment, whereas DLB with RBD (DLBRBD) tends to exhibit more pronounced parkinsonism compared to their counterparts without RBD (Berg et al. 2021; Ferman et al. 2011). However, while nearly all individuals with iRBD progress to PD or DLB, RBD is clinically manifest in only about 50% of patients with PD and 75% of those with DLB (Berg et al. 2021). These observations indicate that the RBD–LBD continuum represents a distinct clinical entity and could offer a unique opportunity to investigate the underlying pathomechanisms throughout the disease trajectory, from prodromal to fully developed stages.

Although the precise pathomechanisms of LBDs remain under investigation, systemic inflammation is recognized as a key factor in the development and progression of the diseases (Amin et al. 2022; Tansey et al. 2022). One potential contributor to systemic inflammation in LBDs is local inflammation driven by microbiota alterations in various parts of the body, particularly the gut (Hirayama et al. 2023; Ryman et al. 2023). Previous studies have shown that PD-associated gut dysbiosis, characterized by the depletion of short-chain fatty acids (SCFA)-producing bacteria and the overgrowth of putative pathobionts, was linked to intestinal barrier disruption, increased immune response, and pathological α-synuclein aggregation (Kleine Bardenhorst et al. 2023; Tan et al. 2022). Microbial composition changes have also been observed in patients with DLB (Nishiwaki et al. 2022) and iRBD (Heintz-Buschart et al. 2018; Heinzel et al. 2021; Huang et al. 2023; Nishiwaki et al. 2020), with some trends overlapping but different from those seen in PD. Additionally, distinct microbiota signatures in oral (Jo et al. 2022; Pereira et al. 2017; Li et al. 2022; Mihaila et al. 2019) and deep nasal (Pal et al. 2021) cavities have been reported in PD. However, these local microbiota alternations do not directly reflect systemic immune and inflammatory responses.

Recently, there has been considerable interest in the existence of a microbiome in the blood of healthy individuals, although its origins and implications remain debated (Castillo et al. 2019; Tan et al. 2023). Of note, the blood microbiome has been repeatedly associated with a variety of diseases, including cardiometabolic diseases, malignancies, and inflammatory and immune diseases (Cheng et al. 2023; Poore et al. 2020; Potgieter et al. 2015). It is speculated that disruption of mucosal barriers in certain disease states may aggravate microbial translocation, resulting in the persistence of microbes in the bloodstream (Cheng et al. 2023; Potgieter et al. 2015). With respect to LBDs, only one study has investigated the blood microbiome characteristics of 45 patients with PD (Qian et al. 2018). They reported higher abundances of the genera *Isoptericola*, *Cloacibacterium*, *Enhydrobacter*, and *Microbacterium* and lower abundances of the genus *Limnobacter* in PD patients compared to controls. However, these findings should be interpreted with caution due to the lack of adjustment for multiple testing, which raises the possibility of false-positive associations. In fact, no significant differences in genera were observed between the two groups after adjusting for multiple testing in that study. Accordingly, the connection between the blood microbiome and PD remains uncertain and requires further investigation. Moreover, there have been no investigations into blood microbiome profiles in patients with DLB or iRBD.

In this study, we aimed to investigate the association between blood microbiome changes and both prodromal and clinically manifest LBDs. To achieve this, we performed 16 S rRNA-based metagenomic analysis on the microbiome profiles of plasma samples from patients within the RBD–LBD continuum, including those with iRBD, PDRBD, and DLBRBD, as well as healthy controls. Additionally, we explored the potential of blood microbiome changes as diagnostic biomarkers and evaluated their relationship with clinical severity across the RBD–LBD continuum.

## Methods

### Study design and participants

We prospectively recruited patients with iRBD, PDRBD, and DLBRBD patients from the Movement Disorders Clinic at the Seoul Metropolitan Government-Seoul National University Boramae Medical Center between 2022 and 2023. All iRBD patients were confirmed by video-polysomnography and were free of parkinsonism and dementia. The exclusion criteria were applied to rule out secondary causes for RBD, as described previously (Lee et al. 2019). The PDRBD group included patients with clinically established PD according to the 2015 Movement Disorders Society (MDS) criteria (Postuma et al. 2015), and the DLBRBD group included patients with probable DLB according to the 2017 revised consensus criteria (McKeith et al. 2017). Phenoconverters from our iRBD cohort within the PDRBD and DLBRBD groups were confirmed to have had iRBD through video-polysomnography prior to their phenoconversion. For the remaining PDRBD and DLBRBD patients, a premorbid history of probable RBD was confirmed using an RBD screening questionnaire score of 6 or higher. On the other hand, healthy controls were recruited from individuals aged 20 to 80 who visited the dental clinic of Seoul National University Bundang Hospital. Blood samples from these individuals were analyzed to establish a reference for normal microbiome signatures. A questionnaire was used to exclude systemic diseases, including hypertension, diabetes, chronic infections, liver, kidney, cardiovascular diseases, autoimmune diseases, malignancies, and antibiotic use within the past 6 months. Electronic medical records, including routine lab results and dental charts, were also reviewed to verify participants’ medical history. The study was approved by the Institutional Review Board of each participating center and conducted in accordance with the Declaration of Helsinki. All participants provided written informed consent before their inclusion in the study.

### Clinical assessment

Overall parkinsonian severity was assessed using the MDS Unified PD Rating Scale (MDS-UPDRS) Parts 1–3. MDS-UPDRS Parts 1 and 2 assess non-motor and motor experiences of daily living, respectively, while Part 3 assesses the severity of parkinsonian motor symptoms. The MDS-UPDRS Part 3 was performed either in a drug-naive state or after at least 12 h of dopaminergic medication discontinuation. Constipation severity was assessed using the MDS-UPDRS Part 1 item 11. Cognitive function tests applied were the Seoul Neuropsychological Screening Battery, including the Mini-Mental State Examination to assess global cognitive performance, the Digit Span Test Forward-Backward and Trail-Making Test (TMT) Part A to assess attention function, the TMT Part B, phonemic and semantic items of the Controlled Oral Word Association Test to assess executive function, the immediate-recall, 20-min delayed recall, and recognition items of the Seoul Verbal Learning Test to assess verbal memory function, and the copying item of the Rey-Osterrieth Complex Figure Test to assess visuospatial function (Ryu and Yang 2023). Raw neuropsychological test scores were converted to standardized z-scores adjusted for age, sex, and educational levels using available normative data (Ryu and Yang 2023). The diagnosis of mild cognitive impairment (MCI) was based on the Level I category of MDS Task Force diagnostic criteria. Impairment on each neuropsychological test was defined as a performance of 1 standard deviation below the norm (Litvan et al. 2012).

### Blood sample collection

Venous blood samples were collected in a 4 mL EDTA tubes under sterile conditions by trained personnel. After centrifugation, plasma aliquots were immediately transported to the lab and stored at − 80 °C until DNA extraction. Microbial DNA was extracted from 1 mL of thawed plasma using the QIAamp DNA Microbiome Kit (QIAGEN, Venlo, Netherlands) following the manufacturer’s protocol.

### 16 S rRNA targeted sequencing

DNA quality was assessed using the Qubit dsDNA HS Assay Kits (Thermo Fisher Scientific Inc., Waltham, MA, USA). PCR targeting the V3 and V4 hypervariable region was performed with the KAPA HiFi HotStart ReadyMix PCR kits (Roche, Basel, Switzerland) according to the manufacturer’s instructions. The primer sequences used for PCR amplification were 519 F:5′-CCTACGGGNGGCWGCAG-3′ and 806R:5′-GACTACHVGGGTATCTAATCC-3′. Libraries were constructed using the NextEra XT DNA library preparation kits (Illumina Inc., San Diego, CA, USA) and pooled at a final loading concentration of 8 pM. Paired-end (2 × 300 bp) sequencing was performed on the MiSeq platform (Illumina).

### Microbiome data analysis

Read preprocessing was performed using a Divisive Amplicon Denoising Algorithm (DADA) 2-based pipeline implemented within the QIIME2 platform (Bolyen et al. 2019; Callahan et al. 2016). In short, amplicon sequencing variants (ASVs) were produced by quality-based filtering and trimming, read deduplication, and ASV inference, followed by paired-end merging and chimera removal. Taxonomic composition was determined by classifying sequences against the 99% SILVA rRNA taxonomy using a pre-trained scikit-learn naïve Bayes classifier implemented with the QIIME q2-feature-classifier plugin (Bokulich et al. 2018; Quast et al. 2013).

The downstream analyses were conducted using R software (ver.4.2.2; R Development Core Team, Vienna, Austria). QIIME artifacts were imported into the R environment via *qiime2* (Bisanz 2018). To adjust for artifactual biases, the ASV table was normalized by rarefaction. Alpha diversity (Chao1) and beta diversity (Bray-Curtis) indices were calculated using the phyloseq package (McMurdie and Holmes 2013). Principal coordinate analysis (PCoA) was applied to visualize overall trends of the sample dissimilarities, whereas permutation multivariate analysis of variance (PERMANOVA) and distance-based redundancy analysis (db-RDA) were conducted to quantify the proportion of variance explained by clinical variables to the total microbial variance using the *vegan* R package (Dixon 2003).

To identify differentially abundant genera between RBD–LBD continuum and healthy control groups, we performed differential abundance (DA) testing using the ANCOM-BC algorithm (Lin and Peddada 2020). Microbial taxa with a prevalence of < 0.05 were excluded prior to analysis. Multiple testing correction was applied using the Benjamini-Hochberg method, with statistical significance set at a *Q-value* < 0.05. This analysis was further extended to examine blood microbiome changes by disease subtypes (iRBD vs. PDRBD vs. DLBRBD) and cognitive status (normal cognition vs. MCI vs. dementia) among patients in the RBD–LBD continuum.

To integrate the univariably associated microbial taxa into a classification model for patients and controls, we performed multivariable logistic regression analyses with L2 (Ridge) regularization using the *glmnet* R package (Friedman et al. 2010). The genus-level abundance table was centered log-ratio (CLR) transformed and filtered based on taxa identified in the DA analysis. Data were randomly split into test and training sets in 10 times repeated 10-fold cross-validation. For each split, the regression model was trained on the training sets and subsequently used to predict the left-out test set. Lambda parameters were selected to minimize mean binomial deviance.

### Statistical analysis

The Shapiro–Wilk test was used to assess the normality of the data. Continuous variables were compared using the Wilcoxon signed-rank test, analysis of covariance, or the Kruskal–Wallis test, and categorical variables were compared using the chi-square test or Fisher’s exact test, as appropriate. When significant differences were detected with analysis of covariance or the Kruskal–Wallis test, post hoc Bonferroni corrections were applied next. CLR transformation was applied to microbial abundance for conventional statistical analyses. Correlations between continuous variables were estimated using the Spearman’s correlation analysis. Multivariable models were evaluated with the *pROC* R package (Robin et al. 2011), with sensitivity, specificity, and area under the receiver operating characteristics curve (AUC) calculated. The 95% confidence interval (CI) for AUC was computed on the 2000 bootstrap replicates. All statistical tests were two-sided, and a *P-value* of < 0.05 was considered to be statistically significant.

## Results

### Clinical and demographic characteristics

A total of 213 participants were initially considered for this study, comprising 44 patients with iRBD, 45 with PDRBD, 24 with DLBRBD, and 100 healthy controls. However, 16 S rRNA gene sequencing could not be successfully generated for 3 patients with iRBD, 4 with DLBRBD, and 6 controls. As a result, 106 samples from patients in the RBD–LBD continuum and 94 samples from controls were included in the final analysis. The demographic and clinical characteristics of the RBD–LBD continuum group and subgroups are shown in Supplementary Table 1. Briefly, the DLBRBD subgroup was older than the PDRBD group; however, there was no significant difference in sex distribution between the subgroups. Based on neuropsychological test results, patients in the RBD–LBD continuum were further classified into three cognitive subgroups: 21 with normal cognition, 65 with MCI, and 20 with dementia. No significant differences were observed among the cognitive subgroups in terms of age and sex (Supplementary Table 2).

### Microbial shifts in the blood of patients across the RBD–LBD continuum

**Fig. 1:**
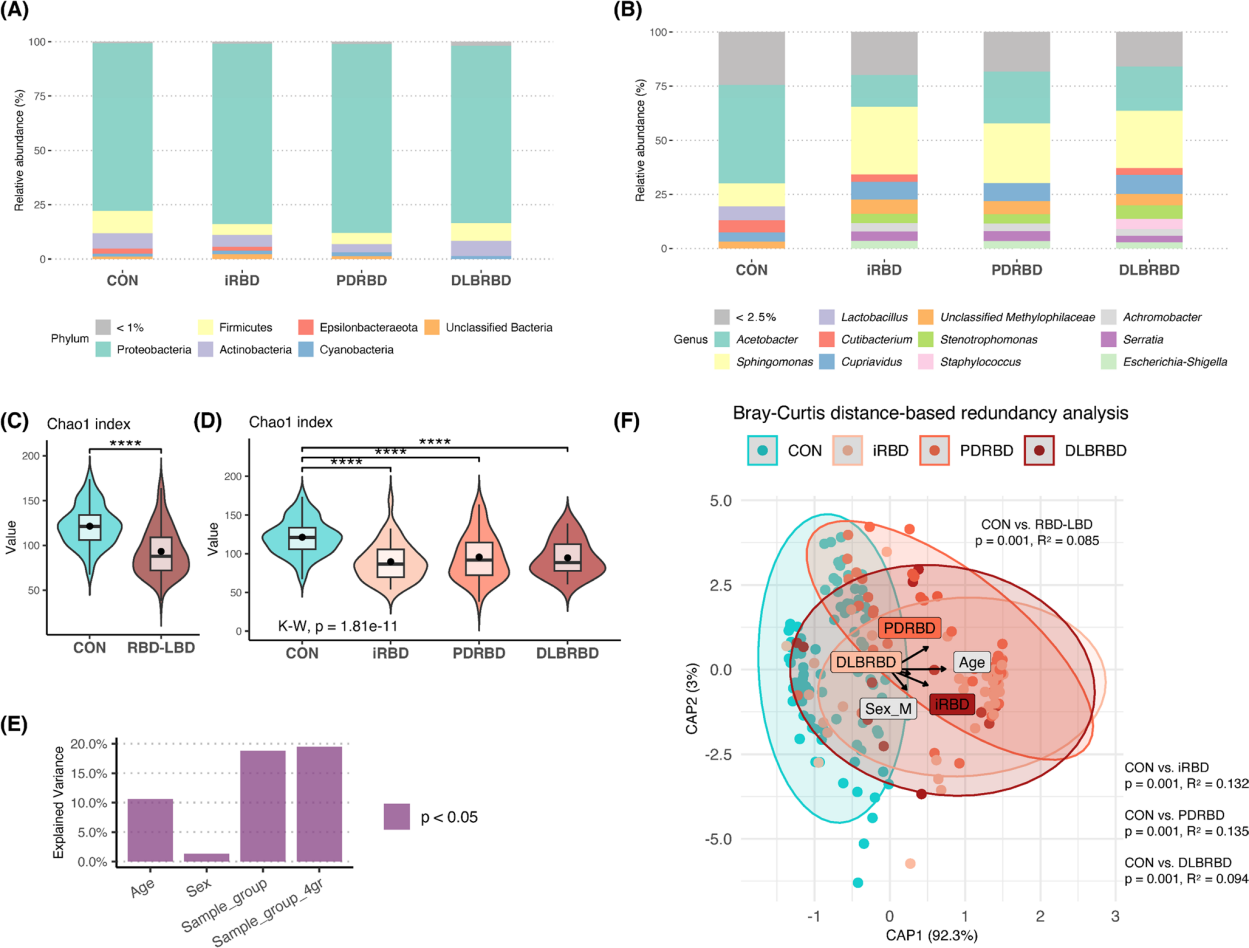
Comparison of blood microbiome between controls and patients within the REM sleep behavior disorder (RBD)–Lewy body disease (LBD) continuum. (**A**) Phylum- and (**B**) genus-level composition of blood microbiome in healthy controls and patients with isolated RBD (iRBD), Parkinson’s disease with probable RBD (PDRBD), and dementia with Lewy bodies with probable RBD (DLBRBD). Phyla and genera representing less than 1% and less than 2.5% of the microbial community are shown as < 1% and < 2.5%, respectively. (**C**, **D**) Violin plots comparing the alpha diversity (Chao1 index) between the control group and the RBD-LBD continuum (**C**) or its subgroups (**D**: iRBD, PDRBD, and DLBRBD). Patients within the RBD-LBD continuum showed significantly lower alpha diversity compared to healthy controls (****p < 0.0001, Wilcoxon; K-W, Kruskal-Wallis test). (**E**) Bar plot representing the associations between beta diversity (Bray-Curtis distance) and subject variables. Bars represent the percentage of explained variance relative to the total microbial variance, and the purple color indicates statistical significance (*p* < 0.05, PERMANOVA). ‘Sample_group’ represents the comparison between the control group and the RBD-LBD continuum, while ‘Sample_group_4gr’ represents the comparison among the control, iRBD, PDRBD, and DLBRBD groups. (**F**) Bray-Curtis distance-based redundancy analysis (dbRDA) of blood microbiome in controls and the RBD-LBD continuum. The colors of the dots and ellipses represent each subject group. Variance constrained by the clinical variables included in db-RDA are illustrated using arrows, where arrow length indicates the contribution of each variable to microbial variance. Significant microbial variance was observed between the control and disease groups (*p* < 0.05, PERMONOVA; adjusted for age and sex). CON, controls

We first analyzed the microbial compositions in the blood of RBD–LBD continuum subgroups compared to healthy controls, focusing on highly abundant bacterial taxa. In all groups, the three most abundant phyla were Proteobacteria, Firmicutes, and Actinobacteria, accounting for 94.7% of the microbiome in controls and 93.5%, 96.0%, and 94.7% in patients with iRBD, PDRBD, and DLBRBD, respectively (Fig. 1A). Among the Proteobacteria, the most dominant genera were *Acetobacter* and *Sphingomonas* across all groups [45.4% and 26.4% (controls), 14.5% and 31.4% (iRBD), 23.9% and 27.6% (PDRBD), and 20.4% and 26.4% (DLBRBD)] (Fig. 1B).

Next, we examined the community-level diversity of blood microbiome in the RBD–LBD continuum group compared with controls to identify disease-specific microbial alterations. Alpha diversity analysis using the Chao1 index revealed lower microbial richness in the blood samples of the RBD–LBD continuum group compared to controls (*P* < 0.001) (Fig. 1C). Similarly, alpha diversity was reduced in the RBD–LBD continuum subgroups relative to controls (*P* < 0.001 for iRBD; *P* < 0.001 for PDRBD; and *P* = 0.003 for DLBRBD; BH-adjusted) (Fig. 1D). However, no significant differences in alpha diversity were found between the iRBD, PDRBD, and DLBRBD subgroups. In beta diversity analysis, age and sex were examined as potential confounders, explaining 10.6% (*P* = 0.001) and 1.3% (*P* = 0.028) of total microbial variance, respectively (Fig. 1E). db-RDA showed distinct clustering of the RBD–LBD continuum and control groups, and PERMANOVA revealed significant microbial composition differences between the groups, even after adjusting for covariates (*P* = 0.001 and R^2^ = 0.085) (Fig. 1F). The differences remained significant when comparing controls to the RBD–LBD continuum subgroups (*P* = 0.001 for all), while there were no significant differences between the subgroups (*P* = 0.829 for iRBD vs. PDRBD, *P* = 0.937 for PDRBD vs. DLBRBD, and *P* = 0.891 for iRBD vs. DLBRBD).

### Microbial taxa associated with the RBD–LBD continuum

**Fig. 2:**
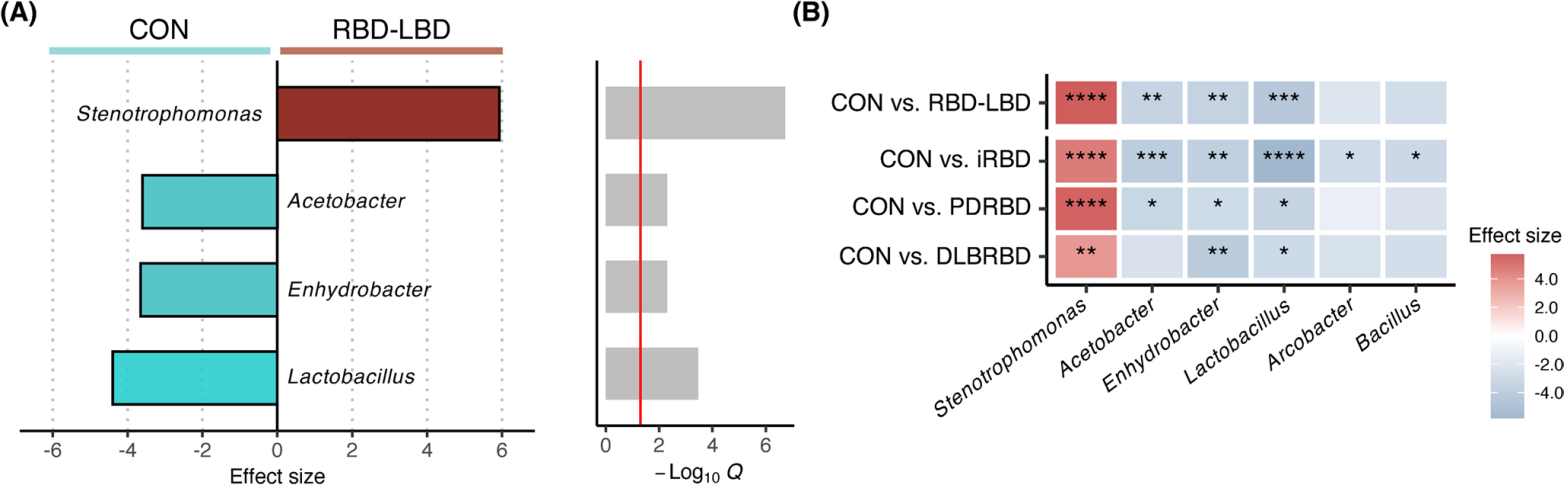
Differentially abundant microbial taxa in the REM sleep behavior disorder (RBD)–Lewy body disease (LBD) continuum compared to healthy controls. (**A**) Bar plots depicting the effect size (left panel) and statistical significance (right panel) of the differentially abundant taxa in the RBD-LBD continuum group. The effect size represents the generalized fold change measured by the ANCOMBC algorithm, with values > 0 indicating taxa increased in patients compared to healthy individuals. The red line in the right panel indicates the threshold for statistical significance (q < 0.05). (**B**) Heatmap illustrating the differentially abundant microbial taxa between the control group and the RBD-LBD continuum subgroups [patients with isolated RBD (iRBD), Parkinson’s disease with probable RBD (PDRBD), and dementia with Lewy bodies with probable RBD (DLBRBD)]. The color scale indicates the effect size, with red representing taxa enriched in the disease groups and blue representing taxa enriched in the control group. Statistical significance is denoted by asterisks (*p < 0.05; **p < 0.01; ***p < 0.001; ****p < 0.0001). CON, controls

DA testing identified specific genera associated with the RBD–LBD continuum (*Q* < 0.05). Given the confounding effects of age and sex on blood microbial variance, as indicated by the beta diversity analysis, we adjusted for these covariates to explore differential genera between the RBD–LBD continuum and control groups. Significant differences were observed in the abundances of four genera in the RBD–LBD continuum group compared to controls: *Stenotrophomonas* was significantly increased, whereas *Lactobacillus*, *Enhydrobacter*, and *Acetobacter* were significantly decreased (Fig. 2A). To identify genera specifically associated with each subtype, we performed DA analysis comparing the iRBD, PDRBD, and DLBRBD subgroups to controls (Fig. 2B). *Lactobacillus* and *Enhydrobacter* consistently decreased across all subgroups relative to controls. *Acetobacter* showed a significant reduction in both the iRBD and PDRBD subgroups, while *Bacillus* and *Arcobacter* were reduced in the iRBD subgroup compared to controls.

### Blood microbiome-based classifiers for predicting the RBD–LBD continuum

**Fig. 3:**
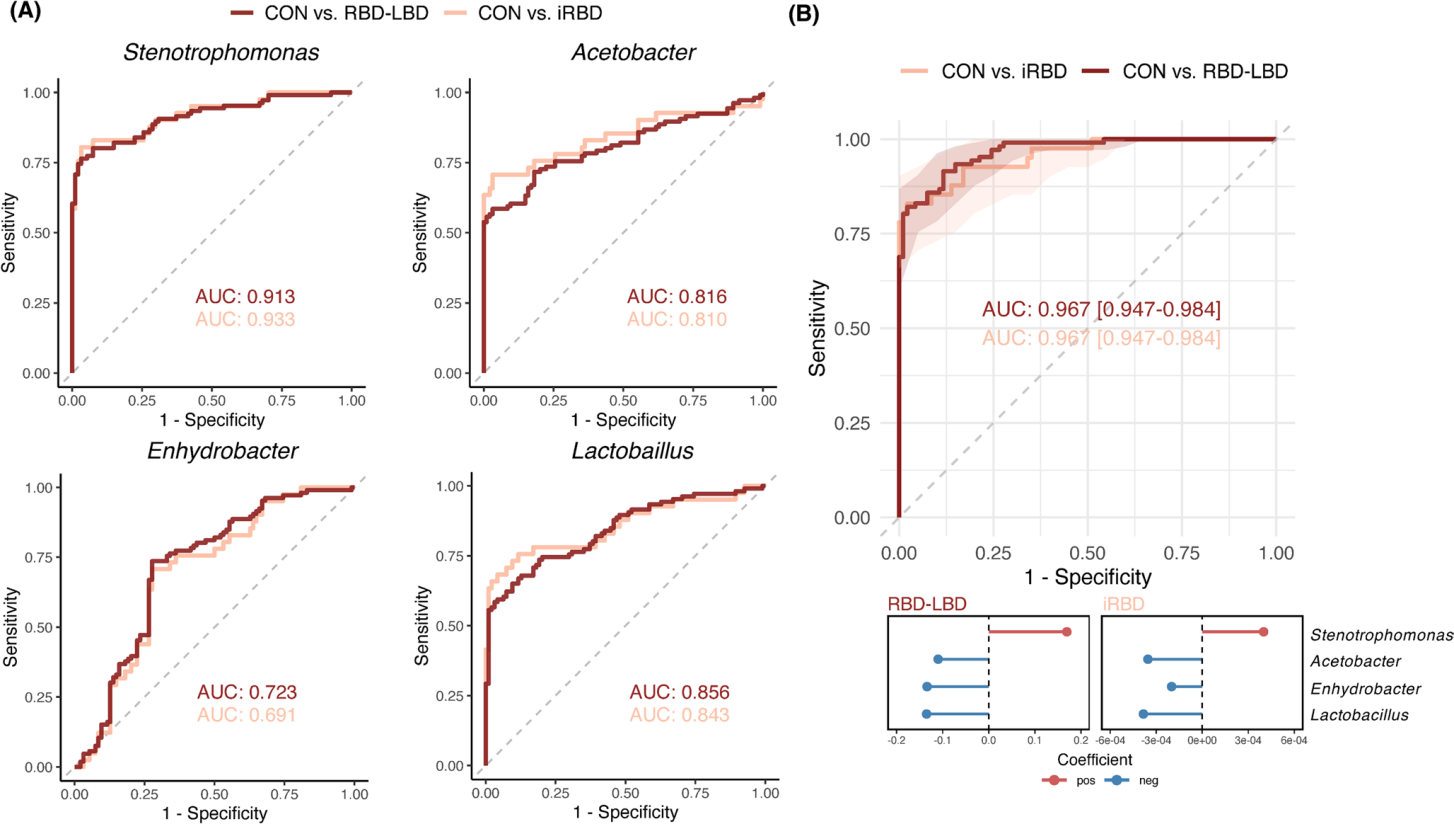
Performance of blood microbiome genera (*Stenotrophomonas*, *Acetobacter*, *Enhydrobacter*, and *Lactobacillus*) in the classification of the REM sleep behavior disorder (RBD)–Lewy body disease (LBD) continuum and healthy controls. (**A**, **B**) Receiver operating characteristic (ROC) curves illustrating the predictive accuracy of each genus as an univariable logistic regression model (**A**) or the combination as a multivariable model (**B**) to classify the RBD-LBD continuum or the iRBD subgroup compared to the control group. The dark red lines represent the performance of each model to discriminate the control group and the RBD-LBD continuum, while the light red lines represent the control group and the iRBD subgroup. Area under curve (AUC) values are provided for each classifier, highlighting their ability to distinguish between the groups. (**B**) Values in parentheses represent 95% confidence intervals (CIs) for the AUC curve. Lollipop plots show the coefficients of variables in the model. Red and blue colors represent positive and negative coefficients, respectively. CON, controls

We evaluated the predictive accuracy of each differentially abundant genus using univariable logistic regression to classify the RBD–LBD continuum and control groups. *Stenotrophomonas*, *Lactobacillus*, *Acetobacter*, and *Enhydrobacter* effectively distinguished between the groups, with high AUC values of 0.917, 0.849, 0.800, and 0.725, respectively (Fig. 3A). To combine microbial taxa with univariable associations into a comprehensive model, ridge logistic regression was used to build a blood microbiome-based classifier for predicting the RBD–LBD continuum. The multivariable model, including *Stenotrophomonas*,* Lactobacillus*, *Acetobacter*, and *Enhydrobacter*, demonstrated high predictive accuracy for the RBD–LBD continuum [AUC = 0.970 (95% CI: 0.950–0.980)] (Fig. 3B). An increase in *Stenotrophomonas* raised the probability of the RBD–LBD continuum, while increases in *Lactobacillus*, *Acetobacter*, and *Enhydrobacter* lowered it. Furthermore, the classifier accurately differentiated the iRBD subgroup from controls, achieving an AUC of 0.956 (95% CI: 0.914–0.987).

### Association between blood Microbiome changes and clinical severity in the RBD–LBD continuum

**Fig. 4:**
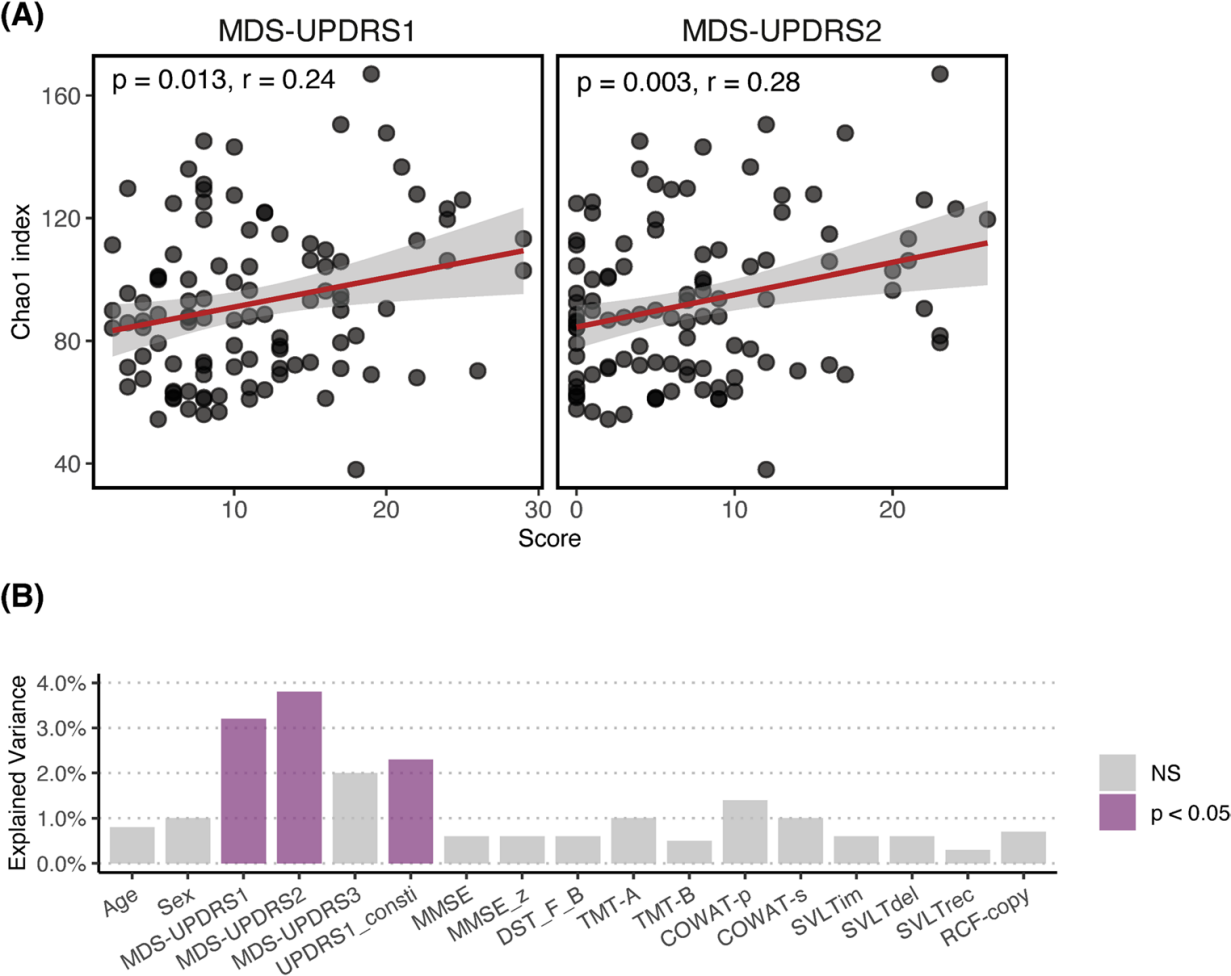
Association between blood microbial alteration and clinicopathological features of the REM sleep behavior disorder (RBD)–Lewy body disease (LBD) continuum. (**A**) Scatter plots showing the correlation between alpha diversity (Chao1 index) and Movement Disorder Society Unified Parkinson’s Disease Rating Scale (MDS-UPDRS) scores. Left panel: MDS-UPDRS Part 1 scores. Right panel: Part 2 scores. The red lines represent linear regression fits, with shaded areas indicating the 95% confidence intervals. (**B**) Bar plot representing the associations between beta diversity (Bray-Curtis distance) and variables. Bars represent the percentage of explained variance relative to the total microbial variance, and the purple color indicates statistical significance (*p* < 0.05, PERMANOVA). Variables include MDS-UPDRS1 (Part 1), MDS-UPDRS2 (Part 2), MDS-UPDRS3 (Part 3), and UPDRS1_consti (MDS-UPDRS Part 1 constipation subscore), as well as cognitive function test scores, including the Mini-Mental State Examination (MMSE), the Digit Span Test Forward-Backward (DST_F_B), Trail-Making Test Part A (TMT-A) and Part B (TMT-B), phonemic and semantic items of the Controlled Oral Word Association Test (COWAT-p and COWAT-s), the immediate-recall, delayed recall, and recognition items of the Seoul Verbal Learning Test (SVLTim, SVLTdel, and SVLTrec), and the copying item of the Rey-Osterrieth Complex Figure Test (RCF-copy)

Alpha diversity index was significantly correlated with MDS-UPDRS Part 1 (Chao 1 index; *R* = 0.21, *P* = 0.034) and Part 2 (*R* = 0.28, *P* = 0.004) scores (Fig. 4A and Supplementary Table 3). Beta diversity measured by Bray-Curtis distance was associated with MDS-UPDRS Part 1 (*R*^2^ = 3.2%, *P* = 0.008) and Part 2 (*R*^2^ = 3.8%, *P* = 0.006) scores, as well as the Part 1 constipation subscores (*R*^2^ = 2.3%, *P* = 0.023) (Fig. 4B and Supplementary Table 4). Within the iRBD subgroup, beta diversity was associated only with the MDS-UPDRS Part 1 constipation subscore (*R*^2^ = 5.7%, *P* = 0.035) (Supplementary Table 5). We did not find significant associations between alpha and beta diversity indices and MDS-UPDRS Part 3 scores or cognitive function tests in the RBD–LBD continuum group (Supplementary Tables 3 and 4). When dividing the RBD–LBD continuum group based on cognitive status, we found no significant differences in alpha or beta diversity among the normal cognition, MCI, and dementia subgroups (Supplementary Fig. 1). Additionally, no differentially abundant genera were identified among these cognitive subgroups.

To further investigate the relationship between blood microbial composition and disease-specific features, such as motor and cognitive function, we analyzed the correlation of specific genera with the MDS-UPDRS Part III, as well as TMT-A and TMT-B, which are among the most reliable cognitive tests for assessing evolving dementia in the RBD-LBD continuum. After adjusting for age and sex, significant correlations were observed between the MDS-UPDRS Part III score and the abundances of *Bradyrhizobium* (*R* = -0.28, *P* = 0.005), *Enhydrobacter* (*R* = -0.22, *P* = 0.027), *Acetobacter* (*R* = -0.23, *P* = 0.021), and *Lactobacillus* (*R* = -0.24, *P* = 0.015); the TMT-A score and the abundances of *Novosphingobium* (*R* = 0.33, *P* = 0.001), *Cutibacterium* (*R* = 0.28, *P* = 0.005), *Acetobacter* (*R* = 0.22, *P* = 0.029), *Corynebacterium* (*R* = 0.25, *P* = 0.014), and *Rhizorhapis* (*R* = 0.22, *P* = 0.028); and the TMT-B score and the abundances of *Acetobacter* (*R* = 0.22, *P* = 0.030) and *Alcanivorax* (*R* = 0.21, *P* = 0.041).

## Discussion

This study provides novel insights into the alterations of the blood microbiome across the RBD–LBD continuum in comparison to healthy controls. Our main findings showed that the blood microbiome divergence between patients in the RBD–LBD continuum and healthy controls was primarily driven by the more abundant genus *Stenotrophomonas* and the less abundant genera *Acetobacter*, *Enhydrobacter*, and *Lactobacillus* in the patient samples. The combination of these genera showed excellent discriminatory ability with an AUC of over 0.9. Of note, similar patterns were observed in the iRBD subgroup alone. To the best of our knowledge, this is the first study to examine the blood microbiome signatures of the RBD–LBD continuum, from the prodromal to the clinically manifest phases of LBD.

At the phylum-level, we found that the proportion of Proteobacteria was increased in the RBD–LBD continuum group. Proteobacteria is a major phylum of Gram-negative bacteria (Stackebrandt et al. 1988) and has been observed to be elevated in both the gut and blood in various chronic inflammatory diseases (Amar et al. 2013; Calandrini et al. 2014; Rizzatti et al. 2017). Among the genera within the Proteobacteria phylum, we found that the *Stenotrophomonas* genus was particularly enriched in patients within the RBD–LBD continuum. While the exact mechanisms linking the *Stenotrophomonas* genus to LBD remain unclear, there are several potential explanations. Notably, *Stenotrophomonas maltophilia* within the *Stenotrophomonas* genus is the only species recognized as a human pathogen (Ryan et al. 2009). This species is commonly detected in pharyngeal swabs from asymptomatic patients, either alone or in combination with other respiratory pathogens, suggesting that its enrichment may originate in the lungs and subsequently spread to the bloodstream. *Stenotrophomonas maltophilia* is known to stimulate peripheral blood monocytes and alveolar macrophages to produce tumor necrosis factor-α, which plays a role in the pathogenesis of airway inflammation. Given the immune-interaction along the lung-brain axis (Azzoni and Marsland 2022; Bajinka et al. 2022; Falsetti et al. 2021; Hosang et al. 2022; Li et al. 2023a; Suman et al. 2022), these inflammatory responses may potentially contribute to central neurodegeneration in LBD. Alternatively, *Stenotrophomonas maltophilia* may simply be well-adapted to thrive in a pro-inflammatory blood environment. As a result, its increased prevalence may reflect the underlying inflammatory state rather than directly contributing to the disease pathology.

Conversely, we observed a reduced abundance of the genera *Acetobacter*, *Enhydrobacter*, and *Lactobacillus* in the blood samples of patients within the RBD–LBD continuum. Some species within *Enhydrobacter* and *Lactobacillus* are known to produce SCFAs (Chalova et al. 2023; Mann et al. 2024). SCFA-producing bacteria play a vital role in maintaining gut health by providing energy to intestinal epithelial cells, strengthening the intestinal barrier, and reducing inflammation (Li et al. 2023b). Moreover, the anti-inflammatory properties of SCFAs support normal microglia development and potentially modulate inflammation in the central nervous system. In line with these findings, a series of studies revealed a lower abundance of SCFA-producing bacteria in the gut of PD patients (Toh et al. 0.^2022^), and their reduced abundance was reported as a potential predictor for the future development of PD in iRBD patients (Nishiwaki et al. 2020). However, a recent meta-analysis found that *Lactobacillus* was frequently enriched in the gut microbiome of PD patients (Romano et al. 2021), which contrasts with our findings in blood samples. Given the broad distribution of *Lactobacillus* across various body sites (Park et al. 2025), this discrepancy suggests that the blood-associated *Lactobacillus* profile likely represents a unique signature originating from multiple anatomical sources rather than exclusively from gut-derived microbiota, as discussed in detail below. Interestingly, supplementation with *Lactobacillus* was shown promote the growth of *Enhydrobacter* in zebrafish models (Hu et al. 2021), indicating a potential interaction between these genera. On the other hand, *Acetobacter* is a genus of acetic acid bacteria. Although the role of *Acetobacter* species in humans is not well understood, they have also been proposed as potential probiotics, similar to *Lactobacillus* (Haghshenas et al. 2015; Wang et al. 2024). Collectively, dysbiosis of such beneficial bacteria may weaken the gut-blood barrier, facilitating microbial translocation and leading to both systemic and central inflammation, ultimately contributing to the pathogenesis of LBDs.

Most prior research on the microbiome in LBDs has focused on the gut microbiome in PD patients, revealing various degrees of alteration compared to healthy controls. However, a previous meta-analysis identified increased abundance of the genera *Akkermansia* and *Bifidobacterium* and decreased abundance of the genera *Roseburia* and *Faecalibacterium* as key features of the PD-associated gut microbiome (Kleine Bardenhorst et al. 2023). These observations differ from the changes identified in the blood microbiome in our study, indicating that the blood microbiome signature may not merely reflect gut dysbiosis. Indeed, evidence suggests that the blood microbiome may derive from multiple sources, including the skin, oral cavity, and nasopharynx, in addition to the gut (Man et al. 2017; Sciarra et al. 2023; Tomás et al. 2012; Wells et al. 1988). Multiple investigations employing 16 S rRNA sequencing demonstrated shared bacterial taxa between blood and these microbiota-rich body regions, prominently featuring genera such as *Corynebacterium*, *Streptococcus*, and *Lactobacillus* (Park et al. 2025). Another study identified substantial overlap of ASVs between blood and a single body site, particularly the skin, within individuals (Rozenberga et al. 2025). Given the distinct microbiome profiles at these sites in PD patients, blood microbiome signature specific to the RBD–LBD continuum observed in this study may represent a composite marker influenced by interactions across various microbial communities. Further studies with concurrent profiling of stool, skin, oral, and nasopharyngeal microbiomes could provide more comprehensive insights into the diversity of blood microbes in the RBD–LBD continuum.

An interesting finding of this study is that beta diversity was associated with MDS-UPDRS part 1 and 2 scores in patients within the RBD–LBD continuum. Although MDS-UPDRS part 2 was developed to assesses motor experiences of daily living, a recent study found that self-reported motor impairments measured using the MDS-UPDRS part 2 reflect both motor and non-motor symptoms (Zolfaghari et al. 2022). Accordingly, our observations suggest that greater variation in blood taxonomic abundance profiles among individuals within the RBD–LBD continuum is more closely associated with non-motor symptoms than with motor symptoms. Specifically, the constipation item of the MDS-UPDRS Part 1 was significantly correlated with beta diversity not only in the overall RBD–LBD continuum group but even in the iRBD subgroup, indicating that the beta diversity in the blood microbiome may be predominantly linked to gastrointestinal symptoms from the prodromal stages of LBD.

The present study has several limitations. First, we did not perform validation with external datasets, which may limit the generalizability of our findings. However, similar trends were observed in individual group analyses, supporting the reliability of the current results, although further validation is necessary. Second, the cross-sectional design of this study precludes establishing causal relationships between blood microbiome changes and the RBD–LBD continuum. Longitudinal studies are warranted to clarify the potential contribution of these microbial alterations to disease progression. Third, we cannot exclude the possibility that unmeasured or unknown confounding factors accounted for the relationships observed in this study. In particular, recent use of antibiotics and immunosuppression may have influenced the blood microbiome, which could not be thoroughly considered in this study due to limited data. Lastly, our analysis was restricted to the genus level in assessing associations with the RBD–LBD continuum. Further investigation using whole metagenome shotgun sequencing is required to explore the blood microbiome at the species or strain level, which could provide deeper insights into the composition of the blood microbiome and its potential role in LBDs.

## Conclusions

This study found blood microbiome signatures specific to the RBD–LBD continuum, characterized by increased abundance of *Stenotrophomonas* and decreased abundance of *Acetobacter*, *Enhydrobacter*, and *Lactobacillus*. The combined microbial profile demonstrated high accuracy in distinguishing patients within the RBD–LBD continuum from controls. These observations highlight the potential of blood microbiome profile as novel biomarkers for the early diagnosis of LBDs. In addition, the presence of these alterations in the prodromal stage of LBDs indicates that blood microbiome changes may be associated with the underlying pathomechanisms of LBD rather than being a consequence of the disease. Further longitudinal studies are necessary to confirm our findings and clarify the role of blood microbial changes in the development and progression of LBDs.

## Data Availability

The raw data that support the findings of this study are available from the corresponding author upon reasonable request.
